# Editorial for Special Issue “Functional Graphene-Based Nanodevices”

**DOI:** 10.3390/nano14050417

**Published:** 2024-02-24

**Authors:** Qijin Cheng, Jian Zhou

**Affiliations:** 1 School of Electronic Science and Engineering, Xiamen University, Xiamen 361005, China; 2 College of Mechanical and Vehicle Engineering, Hunan University, Changsha 410082, China; jianzhou@hnu.edu.cn

As a typical ultra-thin two-dimensional nanomaterial, graphene has many excellent properties, including, but not limited to, mechanical, optical, thermal and electrical properties. For example, it possesses a high Young’s modulus of 1 TPa, a high transmittance of 97.7%, a high thermal conductivity of 5 × 10^3^ W/m.K, and a high carrier mobility of 2 × 10^5^ cm^2^/V.s [1,2,3,4,5]. In particular, owing to its distinct electronic properties, graphene exhibits several electron transport phenomena, including the single-electron tunneling effect, the electron coherence effect, and the anomalous quantum Hall effect [6,7,8,9]. These unique properties endow graphene with a broad range of potential applications in transistors, sensors, photodetectors, energy storage devices, solar cells, transparent conductive electrodes, biomedicine, etc. [10,11,12,13,14,15,16,17,18,19,20]. 

This Special Issue, entitled “Functional Graphene-Based Nanodevices”, focuses on the synthesis, modification and functionalization of graphene-based nanomaterials and their applications in nanodevices. It contains ten articles, as outlined below. 

Graphene-based transistors are considered in contribution 1,2. In contribution 1, the integrated structure of a graphene single-electron transistor and nanostrip electrometer is prepared using the semiconductor fabrication process; the transistor is able to deplete the electrons in the quantum dot structure at low temperatures, while the nanostrip electrometer coupled with the quantum dot can be used to detect the quantum dot signal. In contribution 2, the use of trilayer graphene scrolls in the channel material of field effect transistors for a self-powered tribotronics and mechanosensation matrix is reported; the fabricated transistor is extremely stretchable, has excellent temperature sensitivity and is highly transparent, while the fabricated mechanosensation matrix possesses the tactile sensing properties of high sensitivity (1.125 mm^−1^), a rapid response time (~16 ms), and a durable operation over thousands of cycles. 

Graphene synthesis and its application in heterojunction photodetectors are considered in contribution 3,4. In contribution 3, a remote plasma-enhanced chemical vapor deposition is proposed to directly grow graphene nanowalls (GNWs) at a low radio-frequency power, effectively enhancing the growth rate and reducing the number of structural defects; the fabricated GNWs/HfO_2_/Si photodetector features a low dark current of 3.85 × 10^−10^ A, with a responsivity of 0.19 AW^−1^, a specific detectivity of 1.38 × 10^12^ Jones and an external quantum efficiency of 47.1% at zero bias. Contribution 4 reports a facile approach to the selective synthesis of MoS_2_ on graphene via the application of laser-based photothermal treatment, enabling the direct formation of graphene/MoS_2_ heterostructures; the fabricated graphene/MoS_2_ photodetector is highly responsive to the incident pulsed light, with excellent stability and reproducibility over multiple cycles. 

Graphene-based energy storage devices are considered in contribution 5,6. In contribution 5, a novel structure of lithium-ion capacitors (LICs), using LiBETI as the electrolyte and a self-synthesized graphene/single-walled carbon nanotube composite as the cathode, is successfully assembled; the fabricated LIC has excellent performance, with a specific capacitance of up to 85 F g^−1^, a capacity retention of up to 72% after 10,000 cycles, and a maximum energy density of 182.6 Wh kg^−1^ at a power density of 2678.0 W kg^−1^. In contribution 6, high-performance graphene-based solid-state hydrogen storage devices are created through the mechanical exfoliation of expanded graphite and functionalization with palladium nanoparticles, revealing that the nanoparticle size and dispersion play a crucial role in the uptake and release of hydrogen.

The applications of graphene in sensors are considered in contribution 7,8. Contribution 7 reports a novel crossbeam structure with a graphene varistor protected by Si_3_N_4_ for nano/micro–electro–mechanical system (N/MEMS) mechanical sensors; this structure substantially overcomes the poor reliability of previous sensors with suspended graphene. The fabricated sensor displays excellent performance, with a gauge factor, sensitivity, hysteresis error, nonlinear error, and repeatability error of ~1.35, 33.13 mV/V/MPa, 2.0119%, 3.3622%, and 4.0271%, respectively. In contribution 8, a sensitive and rapid electrochemical method for the detection of paraquat in environmental water samples using a glassy carbon electrode modified with vertically ordered mesoporous silica films and a nanocarbon composite is proposed; the proposed sensor has a superior analytical sensitivity and anti-fouling ability compared with that based on the bare glassy carbon electrode. 

The synthesis and relevant applications of graphene derivatives are considered in contribution 9,10. In contribution 9, the optical characteristics (optical transitions, optical bandgap, absorption coefficient, and absorbance spectrum width) of graphene oxide (GO) and reduced graphene oxide (rGO) are investigated by tailoring the drying time and reduction time at two different temperatures; importantly, the absorption coefficients of the synthesized GO and rGO surpass those reported for exfoliated graphene dispersions by two to three times. In contribution 10, a seeded emulsion polymerization is firstly employed to synthesize polystyrene microspheres, and the uniform monolayer of the polystyrene microspheres is prepared on the substrate via the dipping method; moreover, a single-electron transistor is successfully fabricated based on self-assembled gold nanoparticles and using the polystyrene microsphere template as a mask. 

Overall, this volume provides a collection of selected papers addressing the synthesis and relevant applications of graphene-based nanomaterials, and we hope that the readers find this useful.
